# First Principles Study on the Interaction Mechanisms of Water Molecules on TiO_2_ Nanotubes

**DOI:** 10.3390/ma9121018

**Published:** 2016-12-16

**Authors:** Jianhong Dai, Yan Song

**Affiliations:** School of Materials Science and Engineering, Harbin Institute of Technology at Weihai, 2 West Wenhua Road, Weihai 264209, China; hitdaijh@163.com

**Keywords:** TiO_2_ nanotube, water adsorption, first principles

## Abstract

The adsorption properties of water molecules on TiO_2_ nanotubes (TiO_2_NT) and the interaction mechanisms between water molecules are studied by first principles calculations. The adsorption preferences of water molecules in molecular or dissociated states on clean and H-terminated TiO_2_NT are evaluated. Adsorption of OH clusters on (0, 6) and (9, 0) TiO_2_ nanotubes are first studied. The smallest adsorption energies are −1.163 eV and −1.383 eV, respectively, by examining five different adsorption sites on each type of tube. Eight and six adsorption sites were considered for OH adsorbtion on the H terminated (0, 6) and (9, 0) nanotubes. Water molecules are reformed with the smallest adsorption energy of −4.796 eV on the former and of −5.013 eV on the latter nanotube, respectively. For the adsorption of a single water molecule on TiO_2_NT, the molecular state shows the strongest adsorption preference with an adsorption energy of −0.660 eV. The adsorption of multiple (two and three) water molecules on TiO_2_NT is also studied. The calculated results show that the interactions between water molecules greatly affect their adsorption properties. Competition occurs between the molecular and dissociated states. The electronic structures are calculated to clarify the interaction mechanisms between water molecules and TiO_2_NT. The bonding interactions between H from water and oxygen from TiO_2_NT may be the reason for the dissociation of water on TiO_2_NT.

## 1. Introduction

The demands of energy and the control of environment pollution are factors that inspire us to develop clean and renewable energy sources. Hydrogen is one of the most promising energy carriers due to its high energy density and zero pollution. However, the production of hydrogen with low cost still hinders its application as an energy carrier. Splitting of water on photocatalysts with solar energy is a desirable way to produce hydrogen without pollution and with low-cost. Honda and Fujishima proved photocatalytic hydrogen production from water and demonstrated the decomposition concept using a photo-electrochemical cell [1]. Many studies have been reported in the literature since their pioneering work. Many photocatalysts have been subsequently developed with high quantum efficiency for water splitting under UV and visible light illumination [2,3,4], such as Pt loaded TiO_2_ particle system [5], Au/TiO_2_ [6], platinized SrTiO_3_ single crystals coated with films of NaOH [7], RuO_2_/TiO_2_ [8] and NiO/SrTiO_3_ [9], BaTiO_4_ loaded with RuO_2_ [10], K_4_Nb_6_O_17_ [11], and so on. However, photocatalysts with sufficient band gap positions and high chemical stability for practical applications are still under development. With strongly catalytic activity and high stability, TiO_2_ based semiconductors are one of the promising photocatalysts for water-splitting.

The photogenerated electron-hole pairs of TiO_2_ react with water and produce hydrogen under illumination. However, the hydrogen production rate is hindered by the quick charge recombination and backward reaction [12]. The hydrogen production efficiency is affected by many factors, such as the incorporating of dopants or other materials that affect charge separation and light harvesting [13]. Generally, the photoactivity of cation-doped titania will be decreased due to the increasing recombination of the photoexcited electron–hole pairs [14]. Nowadays, several photocatalysts have been developed. The metal Ag can enhance the hydrogen production using low-energy UV lamps and maintain the hydrogen production of activity-modified TiO_2_ [15]. The Ni, Pd, and Pt can significantly improve the photocatalytic production of H_2_ from water [16]. The metal oxides, such as Ni_2_O_3_ and CuO, are also utilized due to their unique plasmon absorption properties using visible light photo-catalysis [17]. Most recently, the photocatalytic activity of Au@Void@TiO_2_ yolk-shell nanostructures were investigated, in which the thicker and bigger shells displayed higher activity of H_2_ production [18]. For catalysts of TiO_2_ in splitting water, one of the important issues is to understand the interaction mechanisms between water molecules and the TiO_2_ surface in order to improve the photocatalytic activity of TiO_2_. Many works have studied the interactions between a water molecule and the TiO_2_ surface [19,20,21,22,23,24,25,26,27,28,29,30]. It was shown experimentally that water molecules mainly adsorb on the clean TiO_2_(110) surface with a small amount of dissociations [19,20,21]. Controversies exist in the adsorption pathways of water molecules on a TiO_2_ surface under theoretical works. The selections of the coverage of water molecules, slab depth, and even the calculation methods can affect the calculation results [22,23,24,25,26,27,28,29]. Furthermore, defects and impurities greatly affect the hydrolysis efficiency on TiO_2_. The load of Au and Pt can greatly decrease the dissociation barrier of water on TiO_2_ and therefore promote hydrolysis efficiency [30].

The water molecule prefers to adsorb on the top of a five-fold coordinated Ti site with a clean rutile TiO_2_(110) surface with an adsorption energy of 0.92 eV under 1 ML (Mono-Layer) coverage, which is 0.14 eV higher than that of dissociated state [21,31,32]. However, the estimated adsorption energy of water on TiO_2_ by density functional theory (DFT) calculations is sensitive to the parameters used in the DFT calculations, which may be the origin of the discrepancy between different works. The adsorption of water on TiO_2_, especially in the dissociated state, can be significantly enhanced by the presence of fourfold coordinated Ti atoms at the step edge of the surface, due to the easy transfer of charge from the water molecule to the TiO_2_ [31]. The photocatalytic properties of TiO_2_ can be affected by the adsorption of hydroxyl, and thus it is important to understand the interaction mechanisms between the water/hydroxyl and the TiO_2_ surface [33]. There are a variety of distributions of OH groups due to the influence of complex adsorption states of water molecules. Two types of hydroxyls are found to adsorb on the TiO_2_ surface, which could be classified as thermally unstable and thermally stable (isolate hydroxyl groups) in terms of thermostability [33,34,35,36]. The adsorption bands of these hydroxyls are greatly affected by the crystal structure of TiO_2_ and the evacuation temperature. The hydroxyls play different roles in the photo-oxidation process [33], but the chemical environment of hydroxyls on the TiO_2_ surface are still deficient.

Water can adsorb on the TiO_2_ surface in molecular and dissociated states, and therefore, different hydroxyl clusters can be formed [37]. Herman et al. reported the adsorption of water on the anatase TiO_2_(101) surface in a molecular state [38], and observed three desorption states in the Temperature Programmed Desorption (TPD) spectra of the multilayer water, adsorbing in sites of 2-fold-coordinated O and 5-fold-coordinated Ti. Some theoretical calculations are consistent with the above experiments in that the water molecule adsorbs in a molecular state on the (101) surface of anatase TiO_2_ [39], but it will be dissociated on the (001) surface [40,41]. Some theoretical works predicted that water molecules adsorb in rutile TiO_2_ surface in the dissociated state, and the OH group is bonded in the two-coordinated O and five-coordinated Ti sites [42]. The adsorption states are also affected by the defects in the surface of TiO_2_. One O vacancy can capture two OH groups [43,44]. The Highest Occupied Molecular Orbital (HOMO) of the dissociated water on the TiO_2_(101) surface is 0.1 eV higher than that of the molecular state. Therefore, the dissociated state of water on the TiO_2_ has higher photocatalytic activity than that of the undissociated water [45]. Furthermore, the adsorption energy of a water molecule on a rutile TiO_2_(110) surface is only slightly affected by the relative sites and orientations of the water molecule. There are repulsive interactions between neighboring OH groups, causing a decrease of the adsorption energy per H atom from 0.56 eV for isolated OH groups to 0.40 eV for neighboring OH pairs. The twofold-coordinated O is the preferred adsorption site even as the coverage increases up to 1 ML [29].

Due to the large surface area and high efficiency of photocatalytic reactions, TiO_2_ nanotubes (TiO_2_NT) have been attracted great attention since they were discovered in 1996 [46]. The solar water splitting activity of TiO_2_NT increases monotonously with the porosity rather than the wall thickness and/or inner diameter [47]. Theoretical calculations show that the formation of OH is the rate-limiting step for the dissociation of the first water molecule on TiO_2_ nanotube arrays [48]. The adsorption of water on TiO_2_NT is exothermic in both the molecular and dissociated states, and the molecular adsorption is energetically more favorable [49]. However, the influence of interactions between water molecules on adsorption states of water molecules are still not well understood. The adsorption properties of several water molecules on the TiO_2_NT are studied in the present work by first principles calculations. The methods used in this paper are briefly described in Section 2, and the results are presented in Section 3. Conclusions are summarized in Section 4.

## 2. Methodology and Models

The (n, 0) and (0, m) of nanotubes are rolled by the anatase (101) sheet along the [101] and [010] directions, respectively [50]. The numbers of n and m denote the supercell size of the sheet. The sizes of the simulated boxes of the (n, 0) and (0, m) nanotubes (where n = 6 and 9, and m = 3 and 6) are 30.0 × 30.0 × 10.210 Å^3^ and 30.0 × 30.0 × 3.776 Å^3^, respectively [51]. The Vienna ab initio simulation package (VASP) was employed to relax the ions and evaluate the total energy and electronic structures [52,53] with PAW(Projector-Augmented Wave)-GGA (Generalized Gradient Approximation) potential [54]. The self-consistent convergences of the total energy difference and forces are chosen as 0.01 meV and 0.01 eV/Å, respectively. All ions of the TiO_2_ nanotube and adsorbates (water molecule and OH cluster) are allowed to relax to obtain the stable adsorption configurations. The distance between neighboring nanotubes is verified to avoid the interactions between them. The energy cutoff is 400 eV, and 1 × 1 × 2 and 1 × 1 × 8 k-meshes were chosen for the (6, 0) and (0, 3) nanotubes, respectively, to ensure accuracy.

## 3. Results and Discussion

The adsorption energies of the water molecule and OH cluster on TiO_2_NT are evaluated via the following definition:

*E_ads_* = *E_nt+a_* − *E_nt_* − *E_a_* (1)

where *E_nt+a_* and *E_nt_* denote the total energies of the adsorbed water/hydroxyl and clean TiO_2_NTs, respectively. The *E_a_* is the total energy of the adsorbate (water molecule or OH cluster) evaluated using a 1.0 nm × 1.0 nm ×1.0 nm cell.

### 3.1. Adsorption of OH on the Clean TiO_2_NT

**Figure 1:**
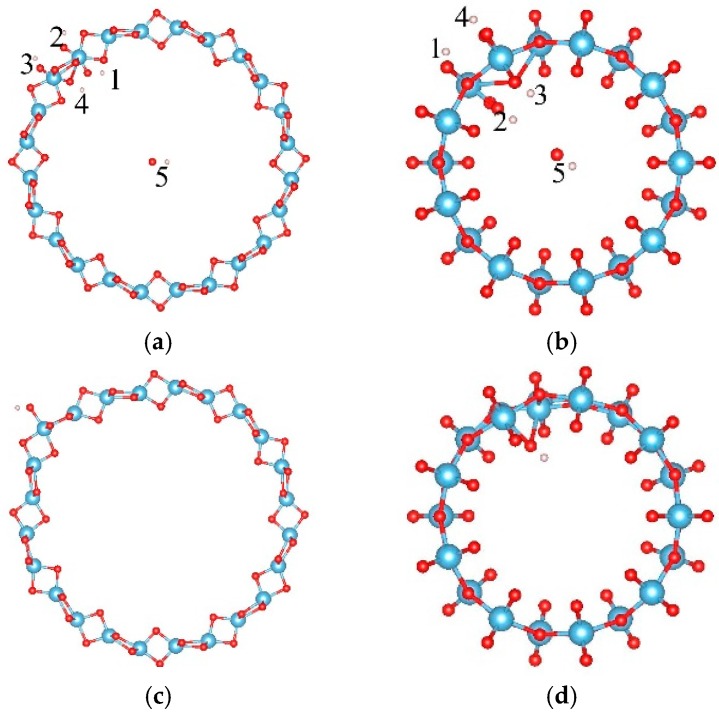
The adsorption sites of OH on the TiO_2_ nanotube. (**a**) The initial adsorption sites of OH on the (0, 6); and (**b**) (9, 0) nanotube, respectively; (**c**) the most stable adsorption sites of OH on the (0, 6) nanotube after full relaxations and (**d**) (9, 0) nanotube, respectively. The blue, red, and white balls denote the Ti, O, and H atoms, respectively.

The adsorption of OH on TiO_2_NT was studied. Five adsorption sites, at the inner ring (labeled as “1” and “4”) and the outer ring (labeled as “2” and “3”), and in the center of the tube (labeled as “5”) are considered as shown in Figure 1a,b. Their adsorption energies are listed in Table 1. For the (0, 6) nanotube, the OH prefers to locate at site “3” and saturates the closest Ti atom after full relaxation with an adsorption energy of −1.163 eV, as shown in Figure 1c. The distance between the oxygen of the OH cluster and its closest Ti atom is 1.915 Å. One Ti atom was dragged out about 0.4 Å by the OH cluster. For the (9, 0) nanotube, the adsorption of the OH cluster also had negative adsorption energy. The most stable adsorption of OH cluster was at site “5” with an adsorption energy of −1.383 eV, and the distance between the OH cluster and the closest Ti atom was 2.111 Å, as shown in Figure 1d. Therefore, the OH cluster was strongly adsorbed on the nanotube by saturating the five-fold Ti ions.

### 3.2. Adsorption of OH on the Hydrogen Terminated TiO_2_NT

**Figure 2:**
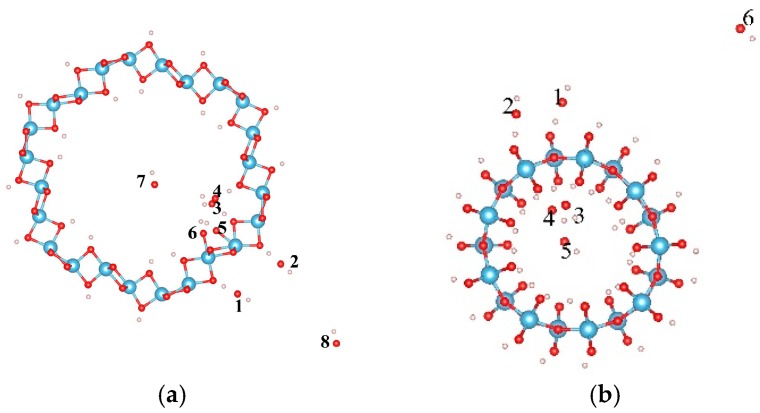
The initial adsorption sites of OH on the H terminated TiO_2_ nanotube. (**a**) (0, 6) and (**b**) (9, 0). The blue, red, and white balls denote the Ti, O, and H atoms, respectively.

**Figure 3:**
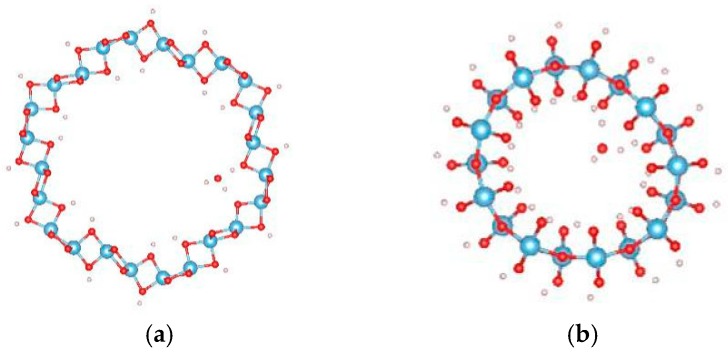
The most stable adsorption sites of OH on the TiO_2_ nanotube after full relaxations; (**a**) (0, 6); and (**b**) (9, 0). The blue, red, and white balls denote the Ti, O, and H atoms, respectively.

The dissociation of water will produce OH and H groups. Thus the adsorption properties of the OH group on the H terminated nanotubes were further studied to clarify the interactions between the OH group and H on TiO_2_NT. Eight and six sites were considered for the adsorptions of the OH on H terminated (0, 6) and (9, 0) nanotubes, respectively. Figure 2 shows the initial adsorption sites, and their adsorption energies after full relaxations are listed in Table 2. For the (0, 6) nanotube, the OH cluster has negative adsorption energy at all considered adsorption sites (around −4.0 eV except at site “8”). The most stable adsorption configuration is when the OH cluster adsorbs on the outer ring of the H terminated (0, 6) nanotube, connecting its O atom with the H atom in the TiO_2_NT, forming a water-like HO–H group with a bond length and angle between the OH and surfacial H of the nanotube of 1.021 Å and 105.8°, as shown in Figure 3a. However, the OH cluster at site “8” is located at the corner of the simulated cell and does not contact the nanotube. The adsorption energy of −0.777 eV may originate from the calculation error of the energy of the OH cluster, and this error can be ignored in terms of the large adsorption energy of the OH cluster in other sites. For the (9, 0) nanotube, the OH cluster at site “6” shows the largest adsorption energy, indicating the strong bonding interactions of the OH cluster with the nanotube. The strongest adsorption of the OH occurs at site “5” with an adsorption energy of −5.013 eV. At site “5”, the OH cluster is initially located at the center of the nanotube (Figure 2b), and moves toward the inner surface, forming a bond with the H atom on TiO_2_NT with its O atom with a bond length of 1.052 Å and a H–O–H angle of 109.5°. A water molecule is therefore formed (Figure 3b). Comparing the adsorption energies of OH on clean and H terminated TiO_2_NTs, one can find that the difference of adsorption energy is mainly caused by the formation of the water molecule. The H termination can greatly affect the adsorption ability of OH on the surface of the nanotube, and therefore, the water-dissociation efficiency will be limited in a H-rich environment due to combinations of OH and H clusters at the surface of the TiO_2_NT.

### 3.3. Adsorption of Water on the Clean TiO_2_NT

The adsorption configurations were investigated by comparing the stability of a water molecule at different adsorption sites of the TiO_2_NTs. It was reported that the adsorption energy of a water molecule on the TiO_2_ surface was almost not affected by the relative site and orientation of the water [45]. Therefore, only several adsorption configurations of a water molecule on TiO_2_NT are considered in the present work. Because the influence of the radius of TiO_2_NT on the adsorption energy of a water molecule is insignificant [48], only the adsorption properties of water molecules on the (0, 3) and (6, 0) nanotubes were studied in the following calculations concerning the computation consuming. It was reported that the adsorption of single water molecules on TiO_2_NT was exothermic and energetically favorable [49]. We also obtained results that the adsorption energies of a single water molecule on the (0, 3) and (6, 0) nanotubes were −0.660 eV and −0.662 eV, respectively. Table 3 shows the adsorption energies of different amounts of water molecules in molecular and dissociated states (denoted as M and D, respectively) on the (0, 3) and (6, 0) nanotubes.

**Figure 4:**
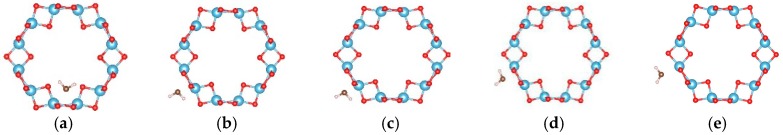
The initial adsorption sites of water molecules on (0, 3) TiO_2_ nanotubes. The blue, red, brown, and white balls denote the Ti, O in TiO_2_NT, O in water, and H atoms, respectively. The indices of (**a**–**e**) denote the different adsorption sites of a water molecule on the TiO_2_ nanotube.

**Figure 5:**
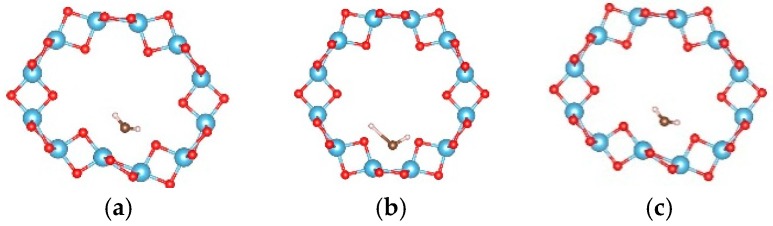
The configurations of a water molecule adsorbed on the (0, 3) TiO_2_ nanotube. (**a**) The relaxed molecular state; (**b**) initial; and (**c**) relaxed configurations of dissociated states. The blue, red, brown, and white balls denote the Ti, O in TiO_2_NT, O in water, and H atoms, respectively.

For the adsorption of a single water molecule on the (0, 3) nanotube, five adsorption sites were considered as shown in Figure 4, and the calculated adsorption energies are listed in Table 3. The five sites have similar adsorption energies, varying in a 0.2 eV energy range. The smallest adsorption energy is −0.660 eV with the relaxed configuration shown in Figure 5a. The oxygen atom of water molecule saturates the five-fold Ti, and the angle between H–O–H is slightly increased to 109.7° by the attraction of the neighbouring oxygen atoms in the nanotube. To further study the stability of this adsorption configuration, the OH and H species of water molecules are initially separated as shown in Figure 5b. After full relaxation, the OH and H species combinated together to the water molecule (as shown in Figure 5c) and the total energy of this configuration is almost identical to that of the most stable adsorption configuration. This result indicates that the single water molecule prefers to adsorb in the molecular state compared to the dissociated mode on the TiO_2_NT.

**Figure 6:**
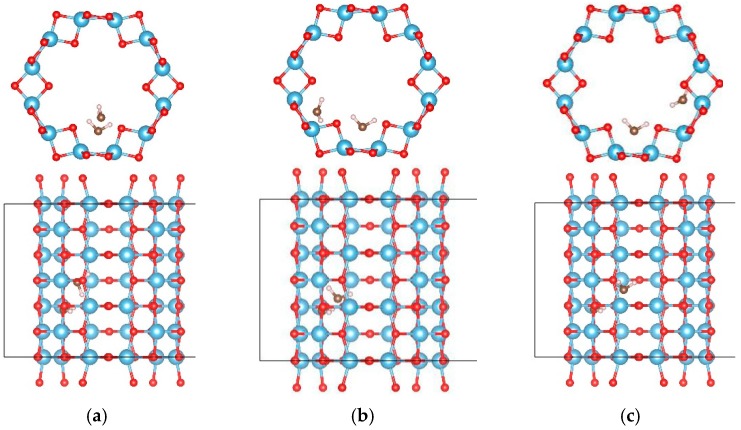
The configurations of two water molecules adsorbed on (0, 3) TiO_2_ nanotubes. The blue, red, brown, and white balls denote the Ti, O in TiO_2_NT, O in water, and H atoms, respectively. The indices of (**a**–**c**) denote the different adsorption sites of a water molecule on the TiO_2_ nanotube.

**Figure 7:**
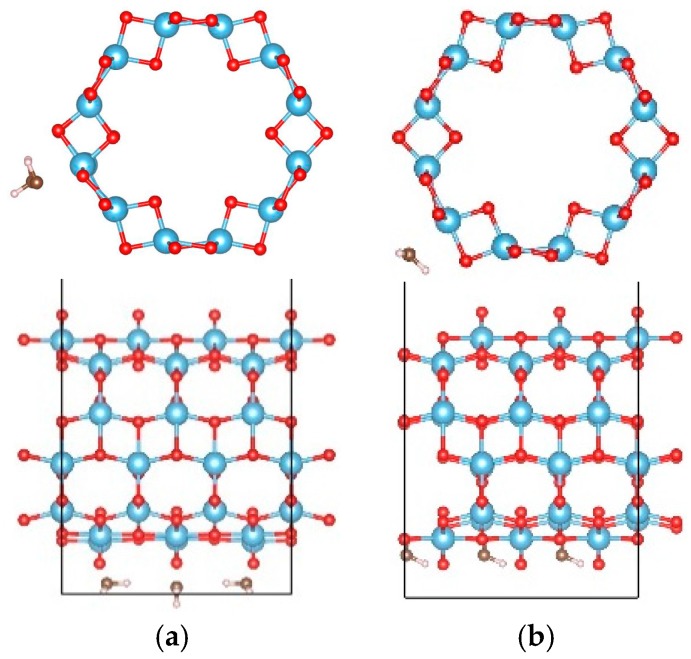
The configurations of three water molecules adsorbed on the (0, 3) TiO_2_ nanotubes. The blue, red, brown, and white balls denote the Ti, O in TiO_2_NT, O in water, and H atoms, respectively. The indices of (**a**,**b**) denote the different adsorption sites of a water molecule on the TiO_2_ nanotube.

**Figure 8:**
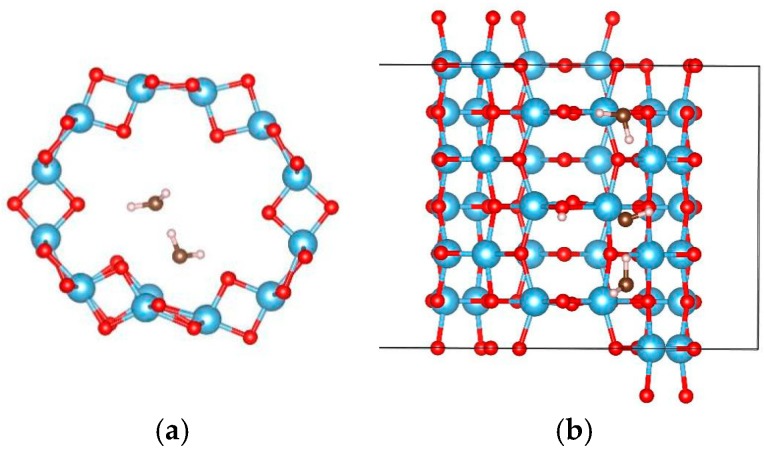
The relaxed configurations of dissociated water molecules adsorbed on the (0, 3) TiO_2_ nanotubes. (**a**) Two water molecules and (**b**) three water molecules. The blue, red, brown, and white balls denote the Ti, O in TiO_2_NT, O in water, and H atoms, respectively.

In order to study the interactions between water molecules, the adsorption of two and three water molecules were studied using a 1 × 1 × 3 supercell of the (0, 3) nanotube, and the adsorption configurations are shown in Figure 6 and Figure 7, respectively. Three and two adsorption configurations for the former and latter cases were considered, respectively. The smallest adsorption energies are −1.128 eV (−0.564 eV/H_2_O) for the former and −1.838 eV (−0.613 eV/H_2_O) for the latter cases. In the multi-water adsorption systems, the relative stabilities between the molecular and dissociated states were also studied by dissociating one water molecule and keeping the others in their adsorption sites as shown in Figure 8. For two water molecule adsorbed system, the initially dissociated OH and H re-combinated together to the molecular state, however, they do not reform a water molecule in the three water molecule adsorbed system. The total energy of the system with two molecular and one dissociated water is 0.032 eV lower than that with three water molecules, indicating that interactions between water molecules prevent the re-combination of the dissociated water molecule.

**Figure 9:**
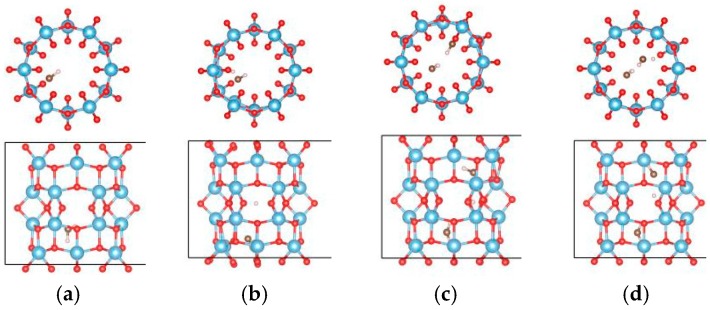
The relaxed configurations of water molecules adsorbed on the (6, 0) TiO_2_ nanotubes. (**a**) One molecular state; (**b**) one dissociated state; (**c**) two molecular states; and (**d**) one molecular state and dissociated state. The blue, red, brown, and white balls denote the Ti, O in TiO_2_NT, O in water, and H atoms, respectively.

**Figure 10:**
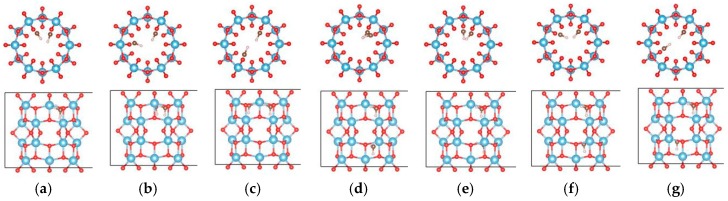
The initial adsorption sites of water molecules on the (6, 0) TiO_2_ nanotubes. The blue, red, brown, and white balls denote the Ti, O in TiO_2_NT, O in water, and H atoms, respectively. The indices (**a**–**g**) denote the different adsorption sites of a water molecule on the TiO_2_ nanotube.

For the (6, 0) nanotube, only one adsorption site for the single water molecule adsorption was studied considering the fact that the adsorption behavior of water is less sensitive to the diameter of the tube [48]. Similar adsorption energies when the water molecule adsorbs at different sites on the (0, 3) nanotube were shown in the above calculations. The estimated adsorption energy of one water molecule on the (6, 0) nanotube is −0.662 eV, which is very close to that of a water molecule adsorbed on the (0, 3) nanotube. The total energy of a single water molecule adsorption on the (6, 0) nanotube in the molecular state (Figure 9a) is about 0.02 eV lower than that in the dissociated state (Figure 9b). In order to clarify the interactions between water molecules, seven different adsorption configurations in a two water molecule adsorbed (6, 0) nanotube are studied, as shown in Figure 10. It also shows about 0.2 eV difference in total energies between the different adsorption configurations. The smallest adsorption energy of the two water adsorbed (6, 0) nanotube is −1.277 eV, which is about 0.15 eV smaller than that of the two water molecules adsorbed on the (0, 3) nanotube. The total energy of the (6, 0) nanotube with one dissociated and one molecular water is about 0.01 eV higher than that of the adsorption of two water molecules in the molecular state, as shown in Figure 9c,d. It is similar to the adsorption of a water molecule on the (0, 3) nanotube. Due to the almost identical adsorption characteristics of water and the OH cluster on the (0, 3) and (6, 0) nanotubes, the adsorption of three water molecules on the (6, 0) nanotube is not considered in the present study. It should be pointed out that although the relative stabilities of dissociated or molecular states are really complex due to the large number of possible adsorption configurations for multiple water molecules on the nanotube, the interactions between water molecules and the nanotube are the key factor determining the adsorption characteristics.

### 3.4. Electronic Structures of Water Adsorbed Nanotubes

**Figure 11:**
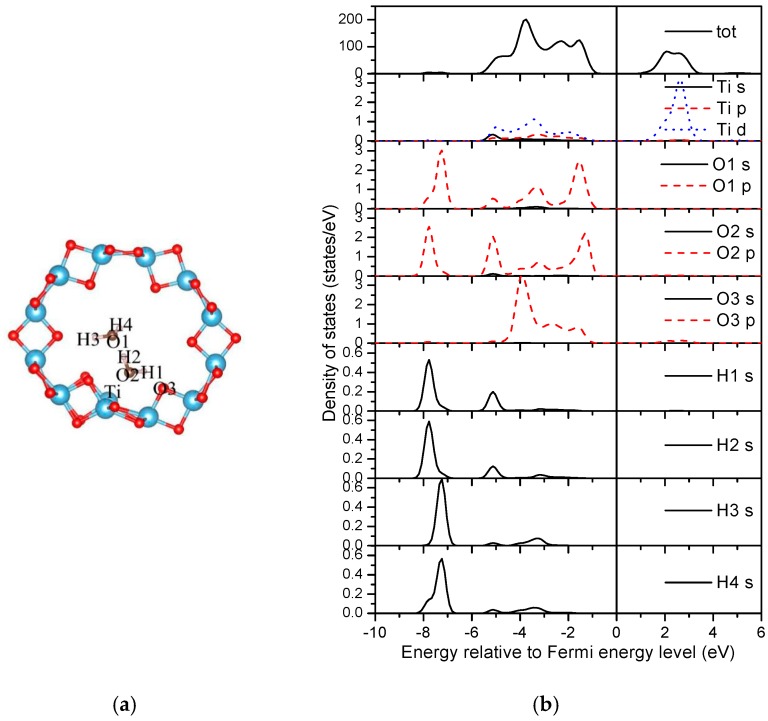
Density of states of the two water molecule adsorbed (0, 3) nanotube. (**a**) Structure; (**b**) DOSs.

The Bader charge analysis was employed to illustrate the charge distributions of atoms in water adsorbed TiO_2_NT [55]. For the clean (0, 3) nanotube, the charges of two-fold and three-fold oxygen atoms are 6.86 e and 7.04 e, respectively, and the value is 2.11 e for Ti, indicating that Ti donates electrons to the oxygen atoms. For the two water molecule adsorbed system (Figure 11a), the Bader charges of the indexed ‘Ti’ and ‘O3’ atoms of the nanotube are 2.06 e and 6.88 e, respectively. The total Bader charges of two water molecules ‘H3–O1–H’ and ‘H1–O2–H2’ are 7.93 e and 7.91 e, respectively. Therefore, a small amount of electrons transfer from the water molecule, especially the H1–O2–H2 molecule, to the nanotube.

**Figure 12:**
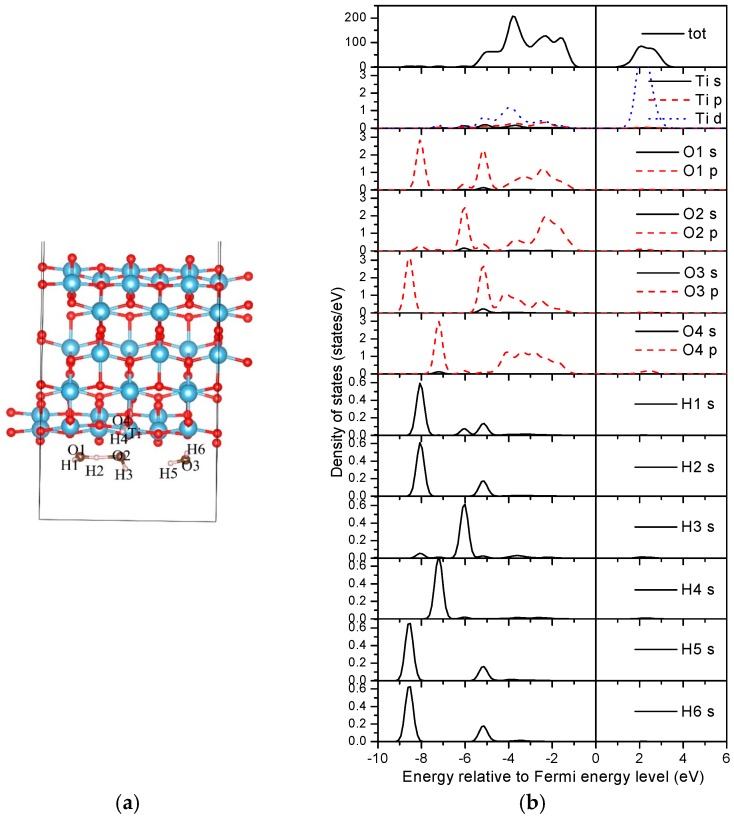
Density of states of a three water molecule adsorbed (0, 3) nanotube. (**a**) Structure; (**b**) DOSs.

For the three water adsorbed nanotube in an energetically stable state (Figure 12a), the Bader charges of the indexed ‘Ti’ and ‘O4’ of the nanotube are 2.04 e and 7.19 e, respectively. The total Bader charges of the H1–O1–H2, H3–O–H4, and H5–O3–H6 ‘molecules’ are 7.94 e, 7.87 e, and 7.91 e, respectively. Specifically, the Bader charge of H4 is about 0.06 e less than that of H3, which may imply a stronger bonding interaction between H4 and its neighboring oxygen atoms. The Bader charge of H2 neighbored by two oxygen atoms is about 0.03 e smaller than that of H1 with one neighboring oxygen atom, causing less charge transfer with respect to H2. The total Bader charge of the H3–O2–H4 ‘molecule’ is the smallest between the considered three water molecules due to the dissociation of H. The “H–O–H–O–H” cluster will then be formed after the dissociation to stabilize the adsorption.

The densities of states (DOSs) are further studied to reveal the interactions between water molecules and the TiO_2_ nanotube. Figure 11b and Figure 12b shown the DOSs of the most stable adsorption configurations of two and three water molecules on the (0, 3) nanotube (Figure 8). It is worth noting that the DFT calculations underestimate the band-gap of the oxides as shown in our calculations. For the two water molecule adsorbed system as shown in Figure 11b, the bonding peaks of H 1*s* are mainly concentrated in the energy region of (−7.0, −8.0) eV, in which they strongly overlap with the oxygen atoms from the neighboring water (O2 *p* orbitals) causing strong bonding interactions. No overlaps between O3 from the nanotube and hydrogen from the water can be detected, implying a vanished weak interaction between them. The O *p* orbitals from water molecules overlap with the Ti *d* orbitals in the energy region of (−5.5, −1.0) eV. Therefore, the adsorptions of water on TiO_2_NT are mainly controlled through the bonding interactions between the oxygen atom in the water molecule and the Ti atom from TiO_2_NT. For the three water molecule adsorbed system, the total DOS of the adsorbed nanotube are similar with that of the two water molecule adsorbed nanotube as shown in Figure 12b. The bonding peaks of the dissociation hydrogen atom (H4) are mainly distributed in the energy region of (−6.6, −7.6) eV, and forms bonds with the oxygen atom (O4) from TiO_2_NT only. Other hydrogen atoms do not escape from the water, i.e., they mainly bond with their neighboring oxygen atoms in water molecules. The ‘Ti’ also slightly bonds with oxygen atoms from water molecules. Therefore, the bonding interactions between the hydrogen from water molecule and the oxygen from the nanotube may be helpful for the water dissociation on TiO_2_NT.

The light irradiation and isoelectronic point are important parameters to evaluate the photocatalysis. Presently, the optical properties, such as the adsorption coefficient, are calculated to evaluate the influence of light on the materials. Most recently, we have calculated the adsorption coefficient of nanotubes along and perpendicular to the tube axis, and achieved similar results with experiments [56]. Our calculations show that the large adsorption coefficients occur in the high energy area (~5.0 eV). The O–H may be easy to dissociate under light irradiation with high energy. Recently, Huang et al. studied the electrochemical phase diagrams combining the density functional calculations and the thermodynamics results [57], in which the experimental chemical potentials for ions in solution were employed. The electrode potentials for the oxidation of water to O_2_ and the reduction of H^+^ to H_2_ were located between the Valence Band Maximum (VBM) and Conduction Band Minimum (CBM) of TiO_2_ under most of pH values. The electronic structures of the nanotube are similar with the bulk TiO_2_, and therefore, the TiO_2_NT should be a promising photocatalyst, as shown by experimental results.

## 4. Conclusions

First principles calculations are carried out to study the adsorption properties of water molecules on TiO_2_NT. Firstly, the adsorption of OH clusters on clean and H-terminated TiO_2_NT are studied. The smallest adsorption energy is about −1.0 eV for OH cluster adsorption on the clean tube and −5.0 eV on the H-terminated tube, respectively. A water molecule will be easily formed for the adsorption of an OH cluster on clean and H-terminated TiO_2_NT. The adsorption properties of single and multiple water molecules on TiO_2_NT are investigated. The single and two water molecules prefer to adsorb in the molecular state, while the dissociated state is more preferable for the adsorption of three water molecules, according to the total energy calculations. Water molecules tend to dissociate into OH and H. The H then bonds with the oxygen from the nanotube, and the OH cluster will form a ‘H–O–H–O–H’ cluster. Therefore, the interactions between molecules can greatly affect the adsorption of water on the TiO_2_ nanotube and may bring about the dissociation of water.

## Figures and Tables

**Table 1:** The adsorption energy of the OH cluster on/in the (0, 6) and (9, 0) nanotubes (eV).

Adsorption Sites	*E_ads_* (eV) on (0, 6)	*E_ads_* (eV) on (9, 0)
1	−0.961	−0.883
2	−0.904	−1.151
3	−1.163	−1.150
4	5.007	−0.388
5	0.104	−1.383

**Table 2:** The adsorption energy, *E_ads_*, of the OH cluster on the H terminated TiO_2_ nanotube (eV).

Adsorption Site	(0, 6)	(9, 0)
1	−4.490	−4.885
2	−4.390	−4.943
3	−4.796	−3.917
4	−4.627	−4.332
5	−4.720	−5.013
6	−4.207	−1.343
7	−4.096	–
8	−0.777	–

**Table 3:** The adsorption energies of water molecules on the (0, 3) and (6, 0) nanotubes (eV).

Adsorption on (0, 3)	*E_ads_* (eV)	Adsorption on 1 × 1 × 3 (0, 3)	*E_ads_*	Adsorption on (6, 0)	*E_ads_* (eV)
1M-1	−0.660	3S-2M-1	−1.128	2M-1	−1.083
1M-2	−0.605	3S-2M-2	−0.887	2M-2	−1.055
1M-3	−0.605	3S-2M-3	−0.964	2M-3	−1.054
1M-4	−0.414	3S-1M-1D	−4.545	2M-4	−1.271
1M-5	−0.411	3S-3M-1	−1.294	2M-5	−1.272
1D	−4.086	3S-3M-2	−1.838	2M-6	−1.272
		3S-2M-1D-1	−5.430	2M-7	−1.277
		3S-2M-1D-2	−5.296	1M	−0.662
		3S-2M-1D-3	−5.264	1D	−4.067
				1M-1D	−4.693

The symbols M and D denote the molecular and dissociated states of water, respectively. The numbers before and after the “M” and “D” indicate the number of water molecules and the adsorption site, respectively. The symbol 3S means the adsorption of water on the 1 × 1 × 3 supercell of (0, 3) nanotube.
